# Supplementary material for: Menthone Inhalation Alleviates Local and Systemic Allergic Inflammation in Ovalbumin-Sensitized and Challenged Asthmatic Mice
Source: Int J Mol Sci. 2022 Apr 4;23(7):4011. doi: 10.3390/ijms23074011 (PMC8999977; doi:10.3390/ijms23074011)
Supplement: Supplementary file 1 [file ijms-23-04011-s001.zip › ijms-1658862-supplementary.pdf]

Supplementary information

Figure S1. Histopathogical findings of lungs were found in the OVA-induced bronchiolitis in mice. Normal architectures of bronchioles of lung in a NSC mouse (A and B, NCL3-2). OVA-induced lungs shown as focal inflammatory cell infiltration, mainly neutrophils and lymphocytes, fairly eosinophils, around perivascular and peribronchial spaces which was graded as slight (2) in DC (C and D, DC3-1), and minimal (1) in PC (E and F, PC3-3); PAS stain. 100 and 400x.

Figure S2. Histopathogical findings of lungs were found in the OVA-induced bronchiolitis in mice. Lungs shown as focal inflammatory cell infiltration, mainly neutrophils and lymphocytes, fairly eosinophils, around perivascular and peribronchial spaces which was graded as slight (2) in ML (A and B, ML3-1), MM (C and D, MM5-1), and minimal (1) in MH (E and F, MH3-1), PAS stain. 100 and 400x.

Table S1. Effects of menthone inhalation with different doses on initial and final body weights, food and energy intakes, as well as feed and energy efficiencies of OVA/Al-sensitized and challenged BALB/c asthmatic mice.

Table S2. Effects of menthone inhalation with different doses on absolute and relative weights of visceral organs of OVA/Al-sensitized and challenged BALB/c asthmatic mice.

Table S3. Effects of menthone inhalation with different doses on serum non-specific IgE、IgA、IgM and IgG antibody titers of OVA/Al-sensitized and challenged BALB/c asthmatic mice.

Table S4. Particular receptors on target cells relating to allergic inflammation.

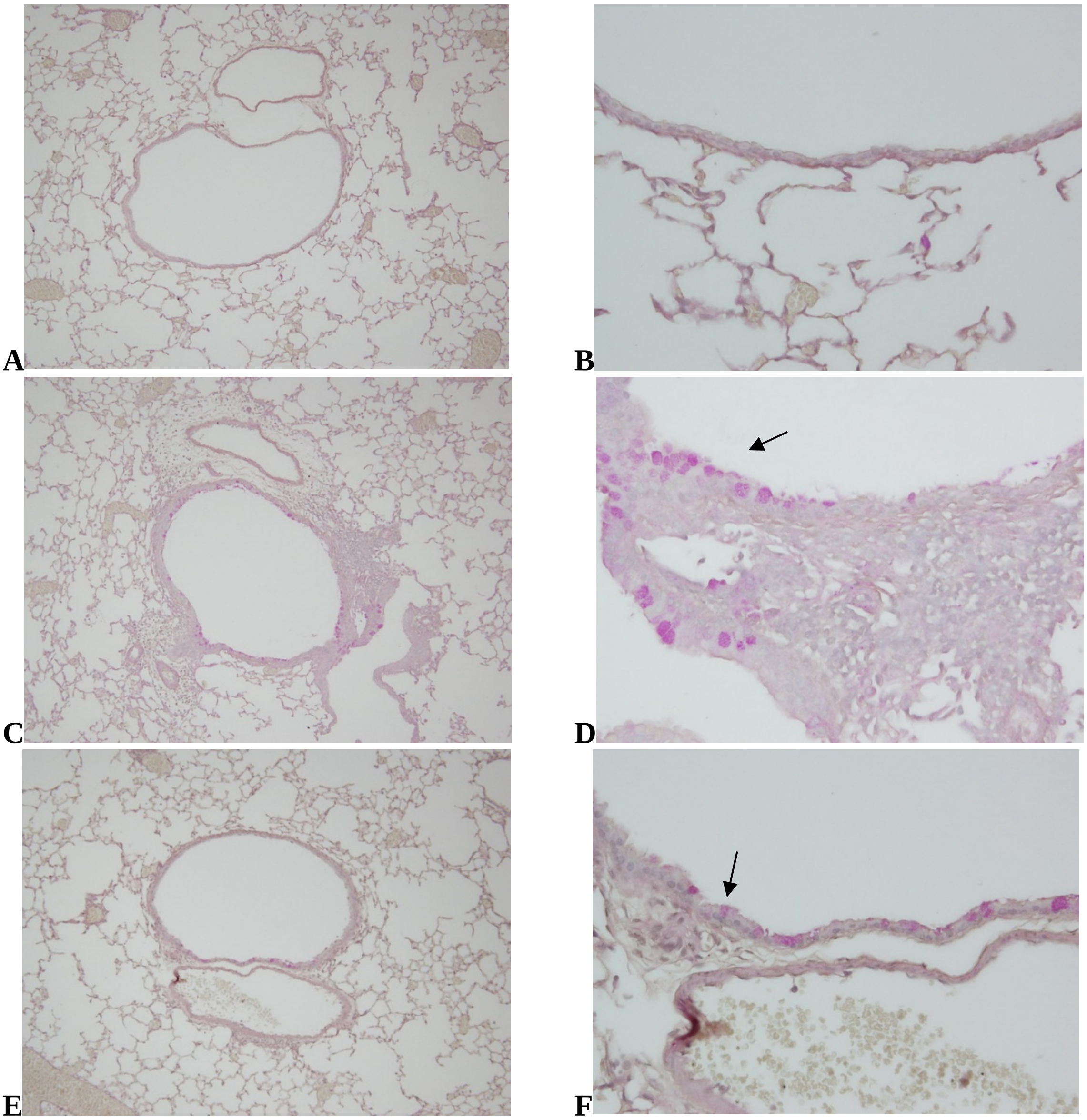

Figure S1. Histopathogical findings of lungs were found in the OVA-induced bronchiolitis in mice. Normal architectures of bronchioles of lung in a NSC mouse (A and B, NCL3-2). OVA-induced lungs shown as focal inflammatory cell infiltration, mainly neutrophils and lymphocytes, fairly eosinophils, around perivascular and peribronchial spaces which was graded as slight (2) in DC (C and D, DC3-1), and minimal (1) in PC (E and F, PC3-3); PAS stain. 100 and 400x.

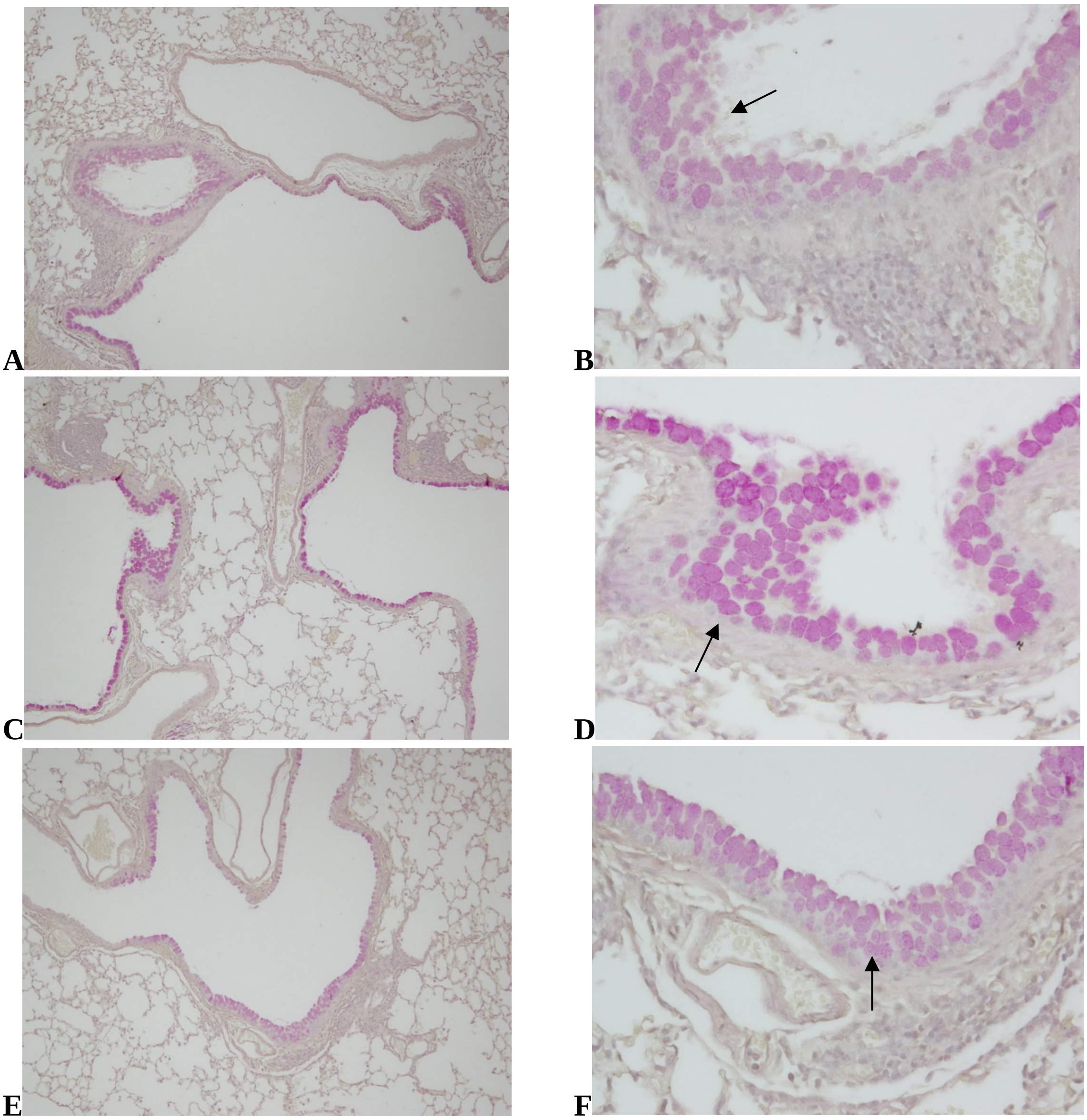

Figure S2. Histopathogical findings of lungs were found in the OVA-induced bronchiolitis in mice. Lungs shown as focal inflammatory cell infiltration, mainly neutrophils and lymphocytes, fairly eosinophils, around perivascular and peribronchial spaces which was graded as slight (2) in ML (A and B, ML3-1), MM (C and D, MM5-1), and minimal (1) in MH (E and F, MH3-1), PAS stain. 100 and 400x.

Table S1

Effects of menthone inhalation with different doses on initial and final body weights, food and energy intakes, as well as feed and energy efficiencies of OVA/Al-sensitized and challenged BALB/c asthmatic mice.

groups	Initial body weight (g)	Final body weight (g)	Body weight gain (g/d)	Feed intake (g/d)	Energy intake (kcal/day)	Feed efficiency (g gain weight/ 100 g feed)	Energy Efficiency (g gain weight/ 100 kcal feed)
DC	21.4 ± 1.2	23.7 ± 1.6	0.10 ± 0.02	4.44 ± 0.32	17.1 ± 1.3	1.59 ± 1.87	0.41 ± 0.49
ML	21.3 ± 1.1	25.0 ± 1.5	0.12 ± 0.03	4.37 ± 0.38	16.8 ± 1.5	2.67 ± 0.65	0.69 ± 0.17
MM	21.3 ± 1.1	24.6 ± 2.0	0.11 ± 0.03	4.25 ± 0.40	16.3 ± 1.6	2.45 ± 0.62	0.64 ± 0.16
MH	21.2 ± 1.1	24.6 ± 1.7	0.11 ± 0.03	4.33 ± 0.51	16.7 ± 2.0	2.53 ± 0.83	0.66 ± 0.22
PC	21.3 ± 1.2	24.4 ± 1.2	0.10 ± 0.04	4.65 ± 0.22	17.9 ± 0.9	2.15 ± 0.97	0.56 ± 0.25
NSC	21.5 ± 1.3	25.5 ± 1.3	0.13 ± 0.04	4.74 ± 0.17	18.2 ± 0.7	2.74 ± 0.88	0.71 ± 0.23

Values are means ± SD (n = 12-15). Values within same column not sharing a common letter are significantly different ($P < 0.05$) from each other assayed by one-way ANOVA, followed by Duncan's multiple range test. There are no significant differences among groups. NSC: non-sensitized control; DC: dietary control; PC: positive control; ML: low dose menthone (0.3 mg/l); MM: medium dose menthone (1.2 mg/l); MH: high dose menthone (6 mg/l).

Table S2

Effects of menthone inhalation with different doses on absolute and relative weights of visceral organs of OVA/Al-sensitized and challenged BALB/c asthmatic mice.

Organ	Group	DC	ML	MM	MH	PC	NSC
liver	ATW (g)	1.22 ± 0.07^{C}	1.23 ± 0.11^{C}	1.22 ± 0.17^{C}	1.21 ± 0.10^{C}	1.41 ± 0.05^{A}	1.31 ± 0.11^{B}
	RTW (%)	5.47 ± 0.09^{B}	5.40 ± 0.13^{B}	5.51 ± 0.35^{B}	5.37 ± 0.24^{B}	6.73 ± 0.33^{A}	5.52 ± 0.33^{B}
kidney	ATW (g)	0.30 ± 0.03^{AB}	0.32 ± 0.01^{AB}	0.32 ± 0.03^{B}	0.34 ± 0.02^{AB}	0.33 ± 0.03^{AB}	0.35 ± 0.04^{A}
	RTW (%)	1.48 ± 0.55^{AB}	1.42 ± 0.10^{B}	1.47 ± 0.10^{AB}	1.50 ± 0.11^{AB}	1.53 ± 0.05^{A}	1.45 ± 0.14^{AB}
thymus	ATW (g)	0.04 ± 0.01^{B}	0.04 ± 0.01^{BC}	0.04 ± 0.01^{BC}	0.03 ± 0.01^{CD}	0.03 ± 0.01^{D}	0.06 ± 0.01^{A}
	RTW (%)	0.19 ± 0.06^{B}	0.16 ± 0.06^{BC}	0.16 ± 0.04^{BC}	0.13 ± 0.04^{C}	0.12 ± 0.05^{C}	0.25 ± 0.05^{A}
epididymal fat	ATW (g)	0.61 ± 0.13^{AB}	0.63 ± 0.11^{AB}	0.54 ± 0.29^{AB}	0.51 ± 0.14^{B}	0.61 ± 0.13^{AB}	0.65 ± 0.10^{A}
	RTW (%)	2.69 ± 0.56^{AB}	2.76 ± 0.44^{AB}	2.38 ± 1.11^{AB}	2.22 ± 0.31^{B}	2.83 ± 0.50^{A}	2.76 ± 0.47^{AB}
spleen	ATW (g)	0.19 ± 0.02^{A}	0.19 ± 0.02^{A}	0.18 ± 0.03^{A}	0.19 ± 0.04^{A}	0.09 ± 0.01^{B}	0.20 ± 0.03^{A}
	RTW (%)	0.85 ± 0.11^{A}	0.81 ± 0.09^{A}	0.82 ± 0.12^{A}	0.86 ± 0.13^{A}	0.42 ± 0.06^{B}	0.82 ± 0.12^{A}
lung	ATW (g)	0.32 ± 0.16	0.29 ± 0.13	0.30 ± 0.13	0.30 ± 0.12	0.29 ± 0.16	0.28 ± 0.10
	RTW (%)	1.43 ± 0.70	1.25 ± 0.51	1.36 ± 0.62	1.30 ± 0.48	1.34 ± 0.70	0.17 ± 0.04

Values are means ± SD (n = 13-15). Values within same row not sharing a common letter are significantly different ($P < 0.05$) from each other assayed by one-way ANOVA, followed by Duncan's multiple range test. NSC: non-sensitized control; DC: dietary control; PC: positive control; ML: low dose menthone (0.3 mg/l); MM: medium dose menthone (1.2 mg/l); MH: high dose menthone (6 mg/l). ATW: absolute tissue weight (g); RTW: relative tissue weight (%).

Table S3

Effects of menthone inhalation with different doses on serum non-specific IgE、IgA、IgM and IgG antibody titers of OVA/Al-sensitized and challenged BALB/c asthmatic mice.

	non-specific antibody levels (μg/ml)				Th1/Th2 antibody ratio	
Groups	IgE	IgA	IgM	IgG	IgG/IgM	IgG/IgE
DC	4.87 ± 1.17^{B}	1334 ± 163^{B}	3443 ± 749	1809 ± 165^{A}	0.53 ± 0.08^{B}	353 ± 49^{B}
ML	5.50 ± 0.35^{A}	1269 ± 446^{B}	3214 ± 1473	1758 ± 239^{A}	0.47 ± 0.07^{B}	315 ± 83^{B}
MM	5.09 ± 0.31^{AB}	1291 ± 211^{B}	3318 ± 944	1726 ± 190^{A}	0.53 ± 0.10^{B}	318 ± 48^{B}
MH	4.58 ± 0.88^{B}	1188 ± 256^{B}	2872 ± 781	1777 ± 188^{A}	0.63 ± 0.13^{A}	406 ± 84^{B}
PC	4.56 ± 0.27^{B}	1948 ± 321^{A}	3429 ± 697	1889 ± 182^{A}	0.55 ± 0.10^{B}	411 ± 47^{B}
NSC	0.39 ± 0.42^{C}	1282 ± 191^{B}	3103 ± 430	612 ± 181^{B}	0.19 ± 0.05^{C}	1913 ± 811^{A}

Values are means ± SD (n = 11-15). Values within same column not sharing a common letter are significantly different ($P < 0.05$) from each other assayed by one-way ANOVA, followed by Duncan's multiple range test. NSC: non-sensitized control; DC: dietary control; PC: positive control; ML: low dose menthone (0.3 mg/l); MM: medium dose menthone (1.2 mg/l); MH: high dose menthone (6 mg/l). Serum dilution: 1 : 100 for IgE detection, 1 : 10000 for IgA detection, 1 : 50000 for IgM detection and 1 : 50000 for IgG detection.

Table S4

Particular receptors on target cells relating to allergic inflammation

receptor	ligands	target cells	known effects after activation
histamine receptor H1 (Hrh1)	histamine	airway smooth muscle, endothelial cells, neurons, dendritic cells, mast cells, monocytes/macrophages, and lymphocytes.	bronchoconstriction, airway inflammation, goblet cell metaplasia, and airway hyperresponsiveness (AHR).
tumor necrosis factor receptor (Tnfr)	TNF-α	many cell types, except red blood cell and resting T cell.	release of pro-inflammatory/chemotactic mediators [regulated on activation, normal T cell expressed and secreted (RANTES), IL-8, and granulocyte-macrophage colony-stimulating factor (GM-CSF)] and up-regulation of adhesion molecules.
epidermal growth factor receptor (Egfr)	transforming growth factor (TGF-α) and heparin-binding EGF-like growth factor (HB-EGF)	epithelial cells of airway.	enhanced expression of mucin genes
chemokine (C-X-C motif) receptor 1 (Cxcr1)	CXCL5, 6, and IL-8	monocyte, neutrophils, endothelial cells, and epithelial cells.	neutrophils, monocytes, and eosinophils chemoattraction.
high affinity receptor for IgE, polypeptide (Fcεr1)	IgE	mast cells and basophiles.	mast cell degranulation

chemokine (C-C motif) receptor 3 (Ccr3)	RANTES, monocyte chemoattractant protein (MCP) - 2, 3, 4, eotaxin and macrophage inflammatory protein (MIP) - 5.	eosinophils, basophiles, mast cell, and airway smooth muscle.	monocytes, eosinophil, basophils, and T cell chemoattraction; activation. and degranulation of eosinophils.
